# Changes in Bioelectrical Impedance Analysis and Lipid Profile in Children Diagnosed with Short Stature Who Undergo Growth Hormone Therapy: One Single-Center Experience

**DOI:** 10.3390/medicina62010209

**Published:** 2026-01-20

**Authors:** Ioana Maria Vlasa, Raluca Monica Pop, Ionut Maxim Vlasa, Ionela Maria Pașcanu

**Affiliations:** 1 Doctoral School of Medicine and Pharmacy, George Emil Palade University of Medicine, Pharmacy, Science, and Technology of Targu Mures, 540142 Targu Mures, Romania; ioana.sintean@gmail.com; 2 Department of Endocrinology, George Emil Palade University of Medicine, Pharmacy, Science, and Technology of Targu Mures, 540142 Targu Mures, Romania; ionela.pascanu@umfst.ro; 3 Department of Endocrinology, Mures County Clinical Hospital, 540139 Targu Mures, Romania; 4 Independent Researcher, 540284 Targu Mures, Romania; vlasaionutmaxim@gmail.com

**Keywords:** short stature, bioimpedance, gender, maternal BMI, lipid profile, rGH treatment

## Abstract

*Background and Objectives*: The effect of growth hormone (GH) on body composition is well recognized, and recombinant human GH (rGH) therapy may improve lean mass and related parameters. The aim of this study was to analyze changes in body composition parameters and lipid profile under rGH treatment in children diagnosed with short stature and to explore potential influencing factors. *Materials and Methods*: A secondary data analysis was conducted in the Endocrinology Department of the Mures County Hospital, Romania, approved by the local Ethics Committee. All children diagnosed with short stature and receiving rGH treatment were eligible for inclusion if they had four body composition analyses at least 6 months apart. Analyzed variables included age, gender, environment, mean rGH dose, height and body mass index (BMI) SDS, body composition parameters assessed by bioimpedance, and family-related variables. Statistical analysis was performed using SPSS v.25 with a level of significance α = 0.05. *Results*: There was no statistically significant trend in body composition parameters taken during serial measurements, except for the sarcopenic index and height (*p* < 0.001). Environment, pubertal development, and family-related variables other than maternal BMI had no significant influence on body composition or lipid profile. Gender differences in body composition revealed that the change in muscle mass (*p* = 0.009) and skeletal muscle mass (*p* = 0.013) was statistically significantly higher for boys, and body fat (*p* = 0.013) for girls. In linear regression analysis, mother’s BMI emerged as a significant predictor for changes in high-density lipoprotein cholesterol (HDL-C) levels (*p* = 0.032, β = −0.691) during rGH therapy. Body composition changes did not differ by treatment indication. *Conclusions*: Gender may be associated with treatment-related changes in body composition during pediatric rGH therapy, while maternal BMI may predict HDL-C variation. rGH treatment appears to improve the sarcopenic index and has minimal and variable effects on the lipid profile.

## 1. Introduction

Short stature is defined by a height more than two standard deviations (SDs) below the mean for a given age, sex, and population group and is estimated to affect 2–3% of the general population [1,2,3,4]. The etiology of short stature is multifactorial, involving genetic, hormonal, and environmental factors [2,3,5]. Endocrine etiologies primarily involve disorders of the growth hormone/insulin-like growth factor I (GH/IGF-I) axis, thyroid dysfunction, cortisol excess, and disturbances in calcium–phosphate metabolism [3,6,7,8].

Recombinant human growth hormone (rGH) therapy is widely used in children experiencing growth failure to enhance linear growth and achieve target height. Treatment with rGH has been approved for a variety of indications, including GHD, children born small for gestational age (SGA), Turner syndrome, Prader–Willi syndrome, and idiopathic short stature (ISS) [9,10,11]. Beyond promoting linear growth, recombinant growth hormone (rGH) therapy exerts multiple metabolic effects, acting directly or via insulin-like growth factor 1 (IGF-1). These effects include modulation of cardiovascular metabolism through its impact on lipid and glucose homeostasis, improvements in body composition, enhancement of muscle and bone mass (musculoskeletal anabolism), and alterations of carbohydrate, lipid, and protein metabolism, as well as body fat distribution [12,13,14,15]. Specifically, rGH therapy stimulates lipolysis via β-adrenergic receptors in adipocytes and inhibits adipocyte maturation. This leads to reductions in total cholesterol (TC), triglycerides (TG), and LDL-cholesterol (LDL-C) while increasing HDL-cholesterol (HDL-C) [16,17,18]. Additionally, rGH therapy may impair glucose tolerance but exerts insulin-like anabolic effects on muscle and bone [12,13,14,15,18,19].

Body composition analysis (BIA) can be performed using several techniques, including bioelectrical impedance, dual-energy X-ray absorptiometry, air displacement plethysmography, magnetic resonance imaging, and computed tomography [20]. Bioimpedance is the most widely used method for measuring body composition, having the following advantages compared to other methods: non-invasive and painless, quick and easy to perform, portable and accessible, low cost, and suitable for repeated measurements. It is performed using bioelectrical impedance devices, which measure the resistance (impedance) of body tissues to a small electrical current, and it is an important tool for assessing the amount and relative proportions of body tissue compartments [21,22,23,24]. Measuring body composition by bioimpedance may include direct or indirect methods. The choice of measurement method depends on whether the focus is clinical or research-related [25,26]. Also, in children, despite differentiated and dynamic growth, body composition indicators can predict future metabolic issues or help monitor the effects of specific treatments [27]. However, in pediatric populations, BIA has important limitations, as its accuracy is strongly influenced by hydration status and may vary according to pubertal stage [21]. Previous studies have reported that rGH replacement in children with growth hormone deficiency (GHD) is associated with favorable changes in body composition, including decreased visceral fat mass and increased lean body mass (LBM) [26]. Nevertheless, data specifically addressing body composition changes in the broader pediatric population with short stature, encompassing both GHD and non-GHD etiologies, remain limited. In particular, evidence regarding BIA-based monitoring of body composition and metabolic parameters in these children is scarce, despite the method’s feasibility and clinical relevance. Therefore, the aim of this study was to analyze changes in body composition parameters and lipid profile during rGH treatment in children with short stature and to investigate potential influencing factors.

## 2. Materials and Methods

### 2.1. Study Design

This study represents a cross-sectional observational study based on secondary data analysis of children diagnosed with short stature who underwent recombinant growth hormone (rGH) treatment at the Endocrinology Department of Mureș County Hospital, Romania, between 2019 and 2024. The analysis was conducted in accordance with the Declaration of Helsinki and was approved by the Ethics Committee of the Mures County Hospital, under approval number 162/23 January 2025. Informed consent was obtained from all subjects involved in the study.

- Inclusion criteria were children over the age of 5 with short stature due to GHD, ISS, SGA, Turner syndrome, or Prader–Willi syndrome who received rGH treatment and had undergone at least four BIA assessments, and each was performed at least six months apart.
- Exclusion criteria were children with other endocrine disorders affecting growth or metabolism (e.g., thyroid dysfunction, hypercortisolism, adrenal insufficiency, late puberty, or disorders of calcium–phosphate metabolism), chronic systemic diseases, incomplete clinical data, fewer than four BIA assessments, irregular follow-up (<6 months), or poor treatment adherence.

### 2.2. Data Collection

The data were gathered from the hospital’s electronic databases and patients’ medical records. For each patient, the following information was collected: demographic and clinical variables (gender, age, environment defined as urban or rural setting, Tanner stage, mean rGH dose), anthropometric data (height and body mass index (BMI)), body composition parameters assessed by BIA (body fat, muscle mass, skeletal muscle mass, phase angle (PhA), and sarcopenic index) and parent-related variables (BMI, educational level, family income). The laboratory parameters analyzed included TC, LDL-C, HDL-C, and TG. All these data were measured at each visit in the morning after a fasting period of at least 12 h. Anthropometric measurements were performed using a Seca stadiometer and a measuring tape (Seca GmbH & Co. KG, Hamburg, Germany). Auxological data obtained was expressed in standard deviations (SDs) using the Romanian Society of Endocrinology’s auxology application, latest available version (https://auxologie.sre.ro/) [28]. The evaluation of pubertal status was performed using a Prader orchidometer (Gima S.p.A, Gessate, Italy) and a 90-degree angle ruler (commercially available). Body composition analysis was performed with the Tanita body composition analyzer model MC-780MA (Tanita Corporation, Tokyo, Japan). All BIA parameters were expressed in percentages, except for the sarcopenic index and phase angle. The sarcopenic index was expressed in kg/m^2^ and was calculated as the appendicular skeletal muscle mass divided by height squared. It helps identify sarcopenia (muscle mass loss) by indicating low skeletal muscle mass adjusted for body size [29]. The phase angle was expressed in degrees (°) and was calculated as the arctangent of reactance divided by resistance, providing a phase shift angle that indicates cell membrane integrity and body cell mass. It reflects the relationship between the body’s resistance (the opposition to electric current flow through body fluids) and reactance (the capacitive opposition caused primarily by cell membranes) [30,31]. All BIA analyses were measured at each visit by the same examiner in the morning after an overnight fast of at least 12 h, in a room with a stable temperature, with each patient being positioned in an orthostatic position on the weighing scale and holding 2 electrodes of the bioimpedance device in their hands. Participants were instructed not to restrict fluid intake and were allowed to consume small amounts of water prior to assessment in order to maintain a normal hydration status. Strenuous physical activity was avoided for 24 h before measurement, and participants were asked to empty their bladder immediately prior to assessment.

### 2.3. Statistical Analysis

Data collection was managed using Microsoft Excel Version 2019 (Microsoft Corporation, Redmond, WA, USA). Statistical analysis was performed using IBM SPSS Statistics for Windows, Version 25.0 (IBM Corp., Armonk, NY, USA). The following statistical tests were employed: the Mann–Whitney test, ANOVA, and the Friedman test to compare central tendencies and assess group differences, Spearman’s rank correlation coefficient to evaluate associations between variables, and multivariate linear regression analysis to emphasize the predictors of the analyzed variables. A two-tailed *p*-value < 0.05 was considered statistically significant. No formal correction for multiple comparisons was applied.

## 3. Results

**Figure 1:**
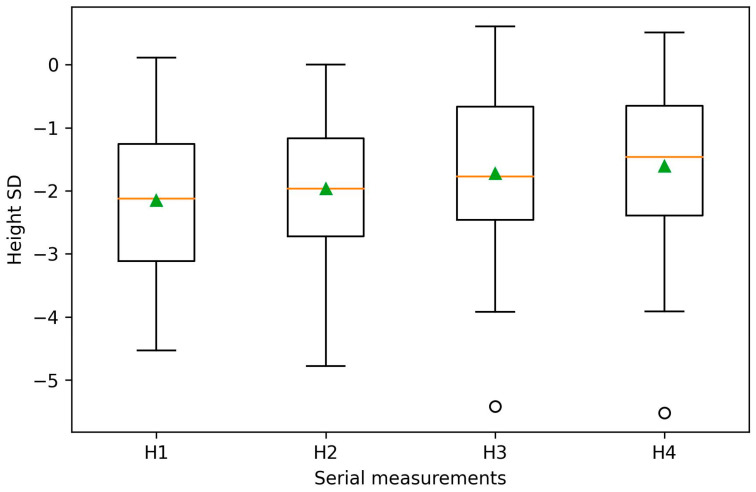
Longitudinal changes in height (H) during recombinant growth hormone (rGH) therapy. Height is expressed as a standard deviation score (SD). Boxplots show the median and interquartile range, with whiskers indicating minimum and maximum values; triangles represent mean values, and circles indicate outliers. H1–H4 correspond to serial height measurements obtained at baseline (H1) and at consecutive follow-up visits (H2–H4).

**Figure 2:**
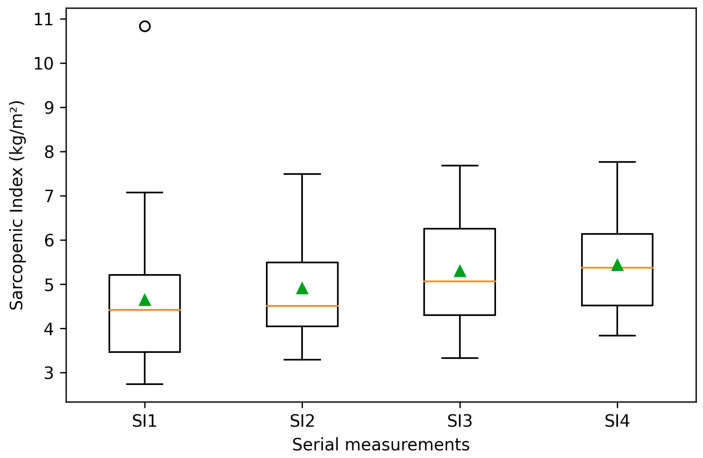
Longitudinal changes in sarcopenic index (SI) during recombinant growth hormone (rGH) therapy. The sarcopenic index is expressed in kg/m^2^. Boxplots show the median and interquartile range, with whiskers indicating minimum and maximum values; triangles represent mean values, and circles indicate outliers. SI1–SI4 correspond to serial sarcopenic index measurements obtained at baseline (SI1) and at consecutive follow-up visits (SI2–SI4).

This study included 30 children diagnosed with short stature who were undergoing rGH treatment. All participants had undergone four BIA assessments, conducted at intervals of at least six months. The etiologies of short stature among the study participants included GHD, ISS, SGA, Turner syndrome, and Prader–Willi Syndrome (Table 1). Demographic and clinical characteristics of the study population are summarized in Table 2. The mean rGH dose was 0.03 mg/kg/day ± 0.04 SD. As expected, height increased significantly during rGH therapy (*p* < 0.001) (Figure 1). Notably, among body composition parameters, a significant longitudinal increase was only observed for the sarcopenic index (*p* < 0.001) (Figure 2), while the remaining parameters did not show statistically significant changes across serial measurements.

During serial measurements, changes in muscle mass (*p* = 0.009) and skeletal muscle mass (*p* = 0.013) were significantly higher for boys, and body fat (*p* = 0.013) for girls (Table 3). After adjustment for age and pubertal stage, no significant sex-related differences were observed. Environment (urban vs. rural) and pubertal status had no significant impact on body composition changes (Table 4 and Table 5).

Participants were grouped according to the etiology of short stature into GHD and non-GHD etiologies, the latter including ISS, SGA, Turner syndrome, and Prader–Willi syndrome. Statistical analysis revealed that the cause of short stature had no significant influence on changes in body composition (Table 6).

Sarcopenic index changes correlated with height gain (r = 0.483, *p* = 0.007) and BMI changes (r = 0.491, *p* = 0.006), while the phase angle correlated with changes in muscle mass (r = 0.488, *p* = 0.006), skeletal muscle mass (r = 0.474, *p* = 0.008), changes in body fat (r = −0.485, *p* = 0.007), and mean rGH dose (r = −0.390, *p* = 0.033) (Table 7). Family income did not show any significant correlation with body composition parameters or anthropometric data.

In multivariate linear regression analyses, changes in height, BMI, and body composition parameters were evaluated as dependent variables, while sex, age, pubertal stage, environment (urban/rural), family income, parental educational level and BMI, and mean rGH dose were examined as predictors; none of these variables emerged as significant predictors.

Comparison of mean lipid values across visits 1–4 showed no statistically significant differences over time for TC, TG, LDL-C, or HDL-C. When stratified by the etiology of short stature into GHD and non-GHD etiologies, the latter, including ISS, SGA, Turner syndrome, and Prader–Willi syndrome, showed that no significant differences in lipid parameter changes were observed between groups, with the exception of HDL-C, which showed a significant improvement in patients with GHD (Table 8).

Triglyceride changes correlated with changes in body fat (r = 0.480, *p* = 0.025), muscle mass (r = −0.442, *p* = 0.015), and skeletal muscle mass (r = −0.415, *p* = 0.023), while cholesterol changes correlated with sarcopenic index changes (r = 0.391, *p* = 0.03). LDL-C changes correlated with changes in the sarcopenic index (r = 0.442, *p* = 0.014) (Table 9). Family income did not correlate significantly with any changes in lipid parameters.

In multivariate linear regression analyses with lipid profile parameters as dependent variables, patient sex, age, pubertal stage, environment (urban/rural), family income, parental educational level and BMI, and mean rGH dose were examined as predictors; among these, maternal BMI emerged as a significant independent predictor of changes in HDL-C during rGH therapy (β = −0.691, *p* = 0.032), whereas no other examined variables showed significant associations.

## 4. Discussion

This study aimed to analyze changes in body composition parameters and lipid profile in children diagnosed with short stature under rGH treatment. Additionally, as a secondary objective, we sought to analyze the role of parental education level, parental BMI, and family income as potential influencing factors.

In the present analysis, rGH therapy was associated with significant longitudinal changes in height and the sarcopenic index. Sex-related differences were observed, and maternal BMI emerged as a potential predictor of lipid profile changes. The improvement in the sarcopenic index suggests a potential beneficial effect of rGH therapy on muscle-related parameters. Socioeconomic variables were not significantly associated with lipid changes in this cohort.

According to the medical literature, growth hormone therapy has been shown to exert beneficial effects on body composition, skeletal development, and lipid metabolism. It promotes increases in muscle and bone mass while contributing to reductions in adiposity, BMI, and lipid levels [13,32,33].

In the present analysis, each performed BIA measured the percentage of body fat, muscle mass, skeletal muscle mass, sarcopenic index, and phase angle. rGH treatment plays an important role in improving body composition by increasing fat-free mass and reducing fat mass. In our study, no statistically significant differences were observed in body composition parameters across serial measurements, except for an improvement in the sarcopenic index, which significantly increased throughout rGH therapy (*p* < 0.001). In contrast with our results, Ryuichi Kuromaru et al. [34] analyzed the long-term effect of rGH treatment on body composition in children with GHD and observed that rGH therapy decreased body fat and increased LBM. A linear growth of lean body mass (LBM) was also observed by P. V. Carroll et al. [35], who compared the continuation or cessation of rGH therapy on BIA in adolescents with severe GHD at completion of linear growth. Another study that analyzed the effect of rGH therapy for one year on BIA parameters in Indian children diagnosed with GHD demonstrated that after the initiation of therapy, there was a significant reduction in body fat and an increase in LBM [36]. Conceptually, the sarcopenic index is used to evaluate the loss of skeletal muscle mass and function. A decrease in the sarcopenic index reflects a decline in muscle fiber size and number, muscle strength, and power [37,38,39]. Even if we did not observe any significant differences between LBM and body fat across serial measurements, the significant improvement of the sarcopenic index may suggest a positive effect of rGH therapy on LBW by improving skeletal muscle mass.

In our study, changes in the sarcopenic index significantly correlated with height gain and BMI changes, while angle phase significantly correlated with changes in muscle mass, skeletal muscle mass, body fat change, and mean rGH dose.

Gender differences in body composition are evident from early childhood and become more pronounced during puberty. For prepubertal children, body fat is typically higher in girls compared to boys, while fat-free mass tends to be higher in boys [40]. In our unadjusted analyses, changes in muscle mass (*p* = 0.009) and skeletal muscle mass (*p* = 0.013) were significantly greater in boys, whereas changes in body fat were significantly greater in girls (*p* = 0.013), in line with findings reported in healthy pediatric populations. However, after adjustment for age and pubertal stage, these sex-related differences were no longer significant, suggesting that maturation-related factors may account for a substantial proportion of the observed differences.

Pubertal status significantly influences body composition, with gender-specific patterns in fat accumulation, muscle development, and bone density [41]. Helena Gleeson et al. [42] highlighted that fat mass and fat-free mass did not change significantly under rGH therapy for prepubertal children diagnosed with severe isolated GH deficiency due to a GHRH receptor mutation, while for postpubertal ones, there was an increase in fat-free mass and a reduction in fat mass. Interestingly, our findings indicate that pubertal development was not significantly associated with changes in body composition. Although Tanner staging was recorded, the timing of pubertal onset is a critical determinant in pediatric growth trajectories, and the limited sample size may have constrained our capacity to conduct robust stratification by pubertal status.

Geographic disparities between urban and rural areas, along with socioeconomic factors, may influence body composition parameters. However, in our sample, the environment had no significant impact on body composition changes. Based on our analysis, parental education and family income were not found to be significantly associated with BIA or anthropometric parameters in the performed analyses.

In the present analysis, there was no significant influence on body composition changes based on the different causes of short stature. In contrast, Pawel Matusik et al. [43] compared body composition parameters in severe, moderate, and no growth hormone deficiency. They found that children with severe GHD had significantly higher fat mass (%) and significantly lower fat-free mass (%) compared to the moderate GHD and no GHD subgroups. No significant differences were observed between the moderate GHD and the no GHD groups. Our results might be explained by the diversity of indications for GH treatment, with some having a very small sample size, lessening the interpretable effect size.

Short stature is often associated with adverse lipid profiles, including higher levels of LDL-C, TC, and TG and lower HDL-C levels. These trends are more pronounced in certain conditions, such as GHD or syndromic causes, like Turner syndrome and Prader–Willi syndrome. In our study, no significant differences were detected in lipid parameters across serial measurements. Overall, an apparent improvement in HDL-C was observed in the GHD subgroup. We observed some correlation between changes in lipids and body composition parameters. The differences in TG significantly correlated with changes in body fat (r = 0.480, *p* = 0.025), muscle mass (r = −0.442, *p* = 0.015), and skeletal muscle mass (r = −0.415, *p* = 0.023), while the sarcopenic index correlated with changes in cholesterol changes (r = 0.391, *p* = 0.03) and LDL-C (r = 0.442, *p* = 0.014). These findings should be interpreted in the context of the exploratory nature of the analysis. These exploratory associations are consistent with studies showing that excess adipose tissue promotes the release of free fatty acid and hepatic triglyceride synthesis, which can impair muscle metabolism and exacerbate lipid abnormalities [44], while muscle tissue plays a critical role in lipid metabolism by enhancing fatty acid oxidation, which may explain this inverse relationship [44,45]. The correlation between TC, muscle mass, and skeletal muscle mass suggests that reductions in muscle mass relative to body size (sarcopenic index) are associated with higher TC levels. This is consistent with findings that sarcopenia contributes to metabolic disturbances, including dyslipidemia [44,46]. In our linear regression analysis with components of lipidic profile as dependent variables, mother’s BMI was a significant predictor for HDL-C changes (*p* = 0.032, β = −0.691). Parental education and family income did not appear to be predictors for changes in lipid profile during treatment with rGH.

This study has several limitations that should be acknowledged. One of the main limitations is its cross-sectional observational design based on secondary data analysis, which confers an exploratory character to the analyses and limits causal inference. In addition, variability in the timing of serial body composition measurements may have influenced the observed results. The relatively small sample size (n = 30) limits statistical power, particularly for subgroup and multivariate analyses, increasing the likelihood of type II errors and reducing the generalizability of the findings. In addition, the absence of correction for multiple comparisons may increase the risk of type I errors. While BIA was selected for its practicality and non-invasive nature, its accuracy in pediatric populations, especially in children with pathological growth conditions, may be influenced by hydration status, pubertal stage, and device-specific calibration. Although all measurements were performed with the same BIA device, under standardized conditions, and by the same trained operator, hydration status was not objectively assessed, and minor variations cannot be entirely excluded. Finally, the inclusion of a varied short stature causes (GHD, ISS, SGA, Turner syndrome, and Prader–Willi syndrome) may obscure condition-specific responses to rGH therapy. This is particularly relevant for syndromic disorders, such as Prader–Willi syndrome, where baseline body composition and treatment responses differ substantially from non-syndromic cases. However, we consider that the main strength of our study is presented by the follow-up of children through four body composition analyses at least 6 months apart and the interest of the topic due to the lack of information on the effects of rGH therapy on body composition parameters and lipid profiles in the pediatric population with short stature.

## 5. Conclusions

Overall, our exploratory analyses suggest that gender may be associated with treatment-related changes, and maternal BMI could be a relevant factor for HDL-C variation in children receiving GH therapy. GH treatment was associated with favorable changes in body composition, particularly the sarcopenic index, while the lipid findings were inconclusive/variable in this sample. These results are preliminary and require confirmation in larger studies, as well as further investigation of the mechanistic links between adiposity, muscle quality, and lipid metabolism to better optimize the balance between growth outcomes and long-term metabolic health.

## Figures and Tables

**Table 1:** Causes of short stature among the study participants (n = 30).

Etiology	N	(%)
GHD	14	46%
ISS	6	20%
SGA	5	17%
Tuner Syndrome	3	10%
Prader–Willi Syndrome	2	7%
Total	30	100%

GHD: growth hormone deficiency; ISS: idiopathic short stature; SGA: small for gestational age; N: number of subjects.

**Table 2:** Demographic and clinical characteristics of the study population (n = 30).

Variable	Value
Mean age	10.08 ± 3.08 years
Gender distribution, N (%)	16 (53) boys 14 (47) girls
Environment distribution, N (%)	14 (47) urban 16 (53) rural

N: number of subjects.

**Table 3:** Median comparison of changes in BIA parameters depending on the patient’s gender.

	Boys	Girls	*p*-Value
Body fat changes, % (IQR)	−0.75 (3.55)	1.55 (4.03)	0.013 *
Muscle mass changes, % (IQR)	1.05 (3.48)	−1.45 (3.9)	0.009 *
Skeletal muscle mass changes, % (IQR)	0.4 (2.05)	−0.9 (2.28)	0.013 *
Sarcopenic index changes, kg/m^2^ (IQR)	0.99 (1.19)	0.81 (0.88)	0.228
Phase angle changes, ° (IQR)	0.05 (0.78)	−0.15 (0.5)	0.269
Height gain, SD (IQR)	0.68 (0.63)	0.48 (1.23)	0.253
BMI changes, SD (IQR)	0.11 (0.96)	0.27 (0.94)	0.480

Mann–Whitney test; SD: standard deviation; IQR: interquartile range; * = *p* < 0.05 (statistically significant).

**Table 4:** Median comparison of changes in BIA parameters depending on the patient’s environment.

	Urban	Rural	*p*-Value
Body fat changes, % (IQR)	0.8 (1.78)	−0.05 (7.78)	0.533
Muscle mass changes, % (IQR)	−0.65 (1.8)	0.2 (7.83)	0.533
Skeletal muscle mass changes, % (IQR)	−0.45 (1.03)	−0.05 (4.48)	0.603
Sarcopenic index changes, kg/m^2^ (IQR)	0.91 (0.89)	0.99 (1.2)	0.454
Phase angle changes, ° (IQR)	−1.1 (0.45)	0.05 (0.8)	0.707
Height gain, SD (IQR)	0.56 (0.8)	0.75 (1.1)	0.151
BMI changes, SD (IQR)	0.27 (0.95)	0.03 (1.02)	0.678

Mann–Whitney test; SD: standard deviation; IQR: interquartile range.

**Table 5:** Median comparison of changes in BIA parameters depending on the patient’s pubertal development.

	Prepubertal (n = 14)	Pubertal (n = 16)	*p*-Value
Body fat changes, % (IQR)	0.70 (5.32)	0.05 (3.42)	0.832
Muscle mass changes, % (IQR)	−0.45 (5.37)	0 (3.6)	0.832
Skeletal muscle mass changes, % (IQR)	−0.40 (3.1)	−0.05 (2.07)	0.832
Sarcopenic index changes, kg/m^2^ (IQR)	0.93 (0.71)	0.94 (0.93)	0.657
Phase angle changes, ° (IQR)	0 (0.67)	−0.05 (2.07)	0.899
Height gain, SD (IQR)	0.67 (0.77)	0.58 (1.3)	0.236
BMI changes, SD (IQR)	0.19 (0.65)	0.11 (0.86)	0.866

Mann–Whitney test; SD: standard deviation; IQR: interquartile range.

**Table 6:** Median comparison of changes in BIA parameters depending on the causes of short stature.

	GHD	Non-GHD	*p*-Value
Body fat changes, % (IQR)	−0.05 (0.31)	0.80 (1.06)	0.261
Muscle mass changes, % (IQR)	0.25 (3.55)	−0.65 (4.8)	0.220
Skeletal muscle mass changes, % (IQR)	−0.05 (1.95)	−0.45 (2.85)	0.279
Sarcopenic index changes, kg/m^2^ (IQR)	0.99 (0.64)	0.84 (0.88)	0.280
Phase angle changes, ° (IQR)	−0.1 (0.45)	0.05 (0.65)	0.786
Height gain, SD (IQR)	0.62 (0.48)	0.59 (1.22)	0.429
BMI changes, SD (IQR)	0.18 (0.58)	0.10 (1.06)	0.480

Mann–Whitney test; SD: standard deviation; IQR: interquartile range.

**Table 7:** Correlation coefficient of body composition parameter changes, auxological data, and average dose of rGH.

	Muscle Mass Changes (%)	Skeletal Muscle Mass Changes (%)	Sarcopenic Index Changes (kg/m^2^)	Phase Angle Changes (°)	Average Dose rGH (mg/kg/Day)
Body fat changes (%)	−0.995 *	−0.998 *	−0.217	−0.485 *	0.167
Trunk fat mass changes (kg)	−0.956 *	−0.957 *	−0.200	−0.480 *	−0.137
Muscle mass changes (%)	-	0.994 *	0.252	0.488 *	0.137
Skeletal muscle mass changes (%)	0.994 *	-	0.230	0.474 *	0.158
Height gain (SD)	0.279	0.247	0.483 *	0.194	−0.390 *
Body mass index changes (SD)	−0.324	−0.328	0.491 *	−0.004	−0.080

Spearman’s rho; SD: standard deviation; * correlation is significant at the 0.05 level (*p* < 0.05).

**Table 8:** Median comparison of changes in lipid parameters depending on the causes of short stature.

	GHD	Non-GHD	*p*-Value
Cholesterol changes, mg/dL (IQR)	−1 (24.75)	3.5 (32.75)	0.190
Triglyceride changes, mg/dL (IQR)	11.5 (23.75)	9.5 (63.75)	0.967
HDL-cholesterol changes, mg/dL (IQR)	−6.5 (15.6)	0.4 (13.62)	0.048 *
LDL-cholesterol changes, mg/dL (IQR)	0.25 (23.13)	7.1 (37.8)	0.533

Mann–Whitney test; IQR: interquartile range; * *p* < 0.05 (statistically significant).

**Table 9:** Correlation coefficient of lipid profile components with body composition parameters.

	Cholesterol Changes (mg/dL)	Triglycerides Changes (mg/dL)	HDL-Cholesterol Changes (mg/dL)	LDL-Cholesterol Changes (mg/dL)
Body fat changes (%)	0.136	0.480 *	0.034	0.075
Trunk fat mass changes (kg)	0.215	0.312	0.079	0.183
Muscle mass changes (%)	−0.124	−0.442 *	−0.024	−0.061
Skeletal muscle mass changes (%)	−0.139	−0.415 *	−0.029	−0.085
Sarcopenic index changes (kg/m^2^)	0.391 *	−0.258	0.347	0.442 *
Phase angle changes (°)	−0.181	0.003	−0.186	−0.016
Average dose rhGH (mg/kg/day)	−0.128	0.039	−0.204	−0.046
Body mass index changes (SD)	0.281	0.326	0.058	0.234
Cholesterol changes	-	−0.124	0.636 *	0.871 *
Triglyceride changes	−0.124	-	0.420 *	−0.149

Spearman’s rho; SD: standard deviation; * correlation is significant at the 0.05 level (*p* < 0.05).

## Data Availability

The data presented in this study are available on request from the corresponding author. Data are not publicly available due to ethical, legal, and privacy restrictions related to sensitive participant information.

## Abbreviations

BIA	Body composition analysis
BMI	Body mass index
GH	Growth hormone
GHD	Growth hormone deficiency
HDL-C	HDL-cholesterol
IGF-1	Insulin-like growth factor I
IQR	Interquartile range
ISS	Idiopathic short stature
LBM	Lean body mass
LDL-C	LDL-cholesterol
PhA	Phase angle
rGH	Recombinant human growth hormone
SD	Standard deviation
SGA	Small for gestational age
SI	Sarcopenic index
TC	Total cholesterol
TG	Triglycerides
